# Signaling Pathways in Thyroid Cancer and Their Therapeutic Implications

**DOI:** 10.14740/jocmr2480w

**Published:** 2016-02-27

**Authors:** Shan Jin, Oyungerel Borkhuu, Wuyuntu Bao, Yun-Tian Yang

**Affiliations:** a Department of General Surgery, Affiliated Hospital of Inner Mongolia Medical University, Hohhot 010050, Inner Mongolia Autonomous Region, China

**Keywords:** Thyroid cancer, Signaling pathway, Targeted therapy

## Abstract

Thyroid cancer is a common malignancy of endocrine system, and has now become the fastest increasing cancer among all the malignancies. The development, progression, invasion, and metastasis are closely associated with multiple signaling pathways and the functions of related molecules, such as Src, Janus kinase (JAK)-signal transducers and activators of transcription (STAT), mitogen-activated protein kinase (MAPK), phosphoinositide 3-kinase (PI3K)/Akt, NF-κB, thyroid stimulating hormone receptor (TSHR), Wnt-β-catenin and Notch signaling pathways. Each of the signaling pathways could exert its function singly or through network with other pathways. These pathways could cooperate, promote, antagonize, or interact with each other to form a complex network for the regulation. Dysfunction of this network could increase the development, progression, invasion, and metastasis of thyroid cancer. Inoperable thyroid cancer still has a poor prognosis. However, signaling pathway-related targeted therapies offer the hope of longer quality of meaningful life for this small group of patients. Signaling pathway-related targets provide unprecedented opportunities for further research and clinical development of novel treatment strategies for this cancer. In the present work, the advances in these signaling pathways and targeted treatments of thyroid cancer were reviewed.

## Introduction

Thyroid cancer is a common malignancy of endocrine system, and its incidence rate is increasing year by year. In 2012, about 56,460 patients were newly diagnosed with thyroid cancer in the United States, resulting in 1,780 deaths. The incidence of thyroid cancer has increased by 4.99 folds from 1989 to 2012. About 62,980 new cases and 1,890 deaths were estimated in 2014 [1, 2]. Since 2002, the incidence rate of thyroid cancer in Korea has been increasing sharply with an annual increasing rate of 24.2% till 2010, and it has now become the most common type of cancer in the country [3]. In Beijing, about 1,099 cases of thyroid cancer were reportedly diagnosed in 2010, yielding an incidence rate of 8.78/100,000. Comparing to the incidence rate in 2001 (2.70/100,000), the incidence rate has increased by 225.2% in the past 9 years with an annual increasing rate of 14.2% [4]. Thyroid cancer has therefore become the most common cancer among all the malignancies. Several treatment modalities including surgical resection, radioactive iodide therapy, and hormone-suppressive therapy could result in good prognosis in most patients with differentiated thyroid cancer (DTC); however, such conventional treatment methods are not effective in treating patients with medullary thyroid cancer (MTC) or anaplastic thyroid cancer (ATC). About 2-5% of patients with DTC lose their differentiated features during treatment or natural course, making them difficult to get effectively treated by radioactive iodide treatment or thyroid stimulating hormone (TSH) suppressive therapy [5]. Distant metastases including pulmonary metastasis (50%), bone metastasis (25%), pulmonary and bone metastasis (20%), and other sites (5%) could be detected in about 10% of the patients and are the major causes of mortality [6]. Although conventional treatment methods are not effective for such patients, researches in molecular biology could bring new chances. The development, progression, invasion, and metastasis of thyroid cancer are closely associated with multiple signaling pathways and functions of related molecules. Any changes in these signaling pathways could be used as biomarkers to help diagnosis and predict the prognosis of thyroid cancer; in addition, potential treatment targets could also be found in these pathways. In the present work, the advances in these signaling pathways and targeted treatments of thyroid cancer were reviewed.

## Src Signaling Pathway

Src family kinase (SFK) is a family of non-receptor tyrosine kinases. Nine members of SFKs have been identified till date including Src, Fyn, Yes, Blk, Fgr, Hck, Lck, Yrk, and Lyn. SFK could be activated by a variety of signals including tyrosine kinase, G protein-coupled receptors, steroid receptor, and signal transducers and activators of transcription (STAT). SFK could then be involved in cell proliferation, growth, motility, migration, angiogenesis, and intracellular transport [7, 8]. Both the direct and indirect activations of SFKs are associated with the progression and metastasis of malignancies. Previous studies have demonstrated that abnormal activation of SFKs is associated with leukemia, small cell lung cancer, non-small cell lung cancer, squamous cell carcinoma of the head and neck (SCCHN), breast cancer, prostate cancer, melanoma, and ovarian cancer. Drugs including dasatinib (BMS-354825), bosutinib (SKI-606), XL-228, KX01 (KX2-391), INNO-406 (NS-187), XL-999, and AZD-0530 which could target Src and SFKs have also been developed [8]. However, only very few studies have investigated the association between Src pathway and thyroid cancer. In an *in vitro* study, Henderson et al used Src inhibitors such as PP2, SU6656, and dasatinib, and the results showed that these inhibitors could effectively inhibit cell proliferation and expression of P-Src and P-FAK in papillary thyroid carcinoma (PTC) cell lines. Dasatinib could also significantly decrease tumor volume in mice carrying RET/PTC1 rearrangement, suggesting that Src pathway plays an important role in regulating PTC cell growth [9]. Another study also found that dasatinib could inhibit the growth of cancer cells, induce apoptosis and cell cycle arrest, and prevent tumor growth and metastasis, which all suggest that Src pathway is very important for the growth and metastasis of thyroid cancer, and Src inhibitors could effectively block thyroid cancer growth and metastasis [10]. SKI-606 could effectively reduce the tumor growth, invasion, and pulmonary metastasis of thyroid cancer in Thrb(PV/PV)Pten(+/-) mice, which could be caused by downregulating the Src pathway and inhibiting the epithelial-mesenchymal transition [11]. Similarly, ADZ0530 could effectively inhibit the cell growth and invasion of PTC and ATC by inhibiting Src-FAK pathway [12]. These findings suggest that the kinases of Src family play critical roles in the signal transduction of the development and progression of thyroid cancer. Activated Src pathway could consequently activate several other pathways including mitogen-activated protein kinase (MAPK), phosphoinositide 3-kinase (PI3K), FAK, and STAT pathways; these activations finally affect the growth, invasion, and metastasis of thyroid cancer [9-13].

## Janus Kinase (JAK)-STAT Signaling Pathway

JAK is a family of non-receptor tyrosine kinases, which could be activated by the combination of cytokines or growth factors to the corresponding receptors. Activated JAK could in turn activate STAT [14, 15]. Four members of JAK family have been identified, which include JAK1, JAK2, JAK3, and TYK2. No individual correspondence exists between JAK and cytokines, which indicates that multiple JAKs could be activated by one cytokine, and several different cytokines could also activate one JAK [16]. STAT is a special family of proteins that could combine with deoxyribonucleic acid (DNA). Seven members of STAT family have been identified including STAT1, STAT2, STAT3, STAT4, STAT5a, STAT5b, and STAT6 [17]. After JAK induces phosphorylation, dimerization of STAT occurs to form STAT dimers, which could enter the cell nucleus and regulate the expression of target genes [18]. STAT3 has been acknowledged as a critical factor of JAK-STAT signaling pathway. Activated STAT3 could increase the expression of bcl-2 and surviving gene, which could in turn reduce the activity of caspase-3 and finally inhibit cell apoptosis [19, 20]. In addition, STAT3 could regulate the expression of genes that mediate survival genes (survivin, bcl-xl, mcl-1, and cellular FLICE-like inhibitory protein), proliferation genes (c-fos, c-myc, and cyclin D1), invasion genes (matrix metalloproteinase-2), and angiogenesis (vascular endothelial growth factor). STAT3 could be involved in the development and progression of tumors by collaborating with other factors including nuclear factor-κB (NF-κB) and hypoxia-inducible factor-1α (HIF1α) [21]. Studies demonstrated that STAT-3 inhibitors could reduce the tumor growth and induce cell apoptosis in SCCHN with activated STAT-3 [22]. Zhang et al [23] investigated STAT3 pathway in 49 patients with PTC (22 with and 27 without lymphatic metastases), and they found that the level of STAT3 expression in benign, non-neoplastic tissue is barely detectable. However, STAT3 expression was found in 11 of the 35 (31%) thyroid cancer tissues; pSTAT3 expression was found in only three of the 35 (9%) benign tissues, but in 40 of the 41 (98%) of the cancer tissues. In addition, pSTAT3 was detected in only four of the 22 (18%) lymph nodes from patients without lymphatic metastases, but in 12 of the 19 (59%) lymph nodes from patients with lymphatic metastases, among which, 45% were with strong staining, suggesting STAT3 pathway as a ubiquitous in PTC, which could activate pSTAT3 and promote the metastasis of PTC. Level of phospholipase D2 was found significantly higher in human PTC tissues than in normal tissues, which could collaborate with thyroid oncogenic kinase RET/PTC to activate STAT-3 [24]. High-fat diet was used to successfully induce ATC in Thrb(PV/PV)Pten(+/-) mice in a study by Kim et al, which suggested that high-fat diet induced the expression of two target genes of STAT3, namely, cyclin D1 and phosphorylated retinoblastoma protein encoding genes via JAK2-STAT3 pathway. STAT3 could thus promote the effects of obesity induced by high-fat diet on the development and progression of thyroid cancer in Thrb(PV/PV)Pten(+/-) mice [25]. These findings suggested the involvement of JAK-STAT3 pathway in the development, progression, and metastasis of thyroid cancer; however, Couto et al [26] found that the expression of tyrosine-phosphorylated or activated STAT3 (pY-STAT3) could be detected in patients with PTC (63/110, 57%), and the level of pY-STAT3 was negatively correlated with the size and distant metastasis of the tumors. STAT3 gene knock-out resulted in downregulation of multiple transcripts including tumor suppressor insulin-like growth factor binding protein 7; in addition, increase in glucose consumption, lactate production, and expression of HIF1α target genes were found after STAT3 gene knock-out, suggesting STAT3 as a negative regulator of aerobic glycolysis. Therefore, the researchers have suggested that STAT3 could also be a negative regulator of tumor growth [26], which is in accordance with the findings of Sosonkina et al [27]. Their research has also suggested that JAK/STAT3 pathway could only inhibit but not promote thyroid cancer [27]. In summary, the biological effects of JAK/STAT3 on thyroid cancer are very complicated; further studies are needed to clarify the associations between JAK/STAT3 pathway and thyroid cancer to help develop novel drugs for targeted therapy.

## MAPK Signaling Pathway

MAPK is a family of intracellular serine/threonine protein kinases with four parallel MAPK signaling pathways identified in mammalian cells including extracellular-signal-regulated kinase (ERK), c-Jun N-terminal kinases (JNK/SAPK), p38MAPK, and ERK5/BMK1 [28, 29]. MAPK pathway could be activated by receptor tyrosine kinase, G protein-coupled receptors, and cytokines. It can then transduce extracellular signals into the cells and nuclei to induce biological responses including cell proliferation, differentiation, and apoptosis. Overactivation of MAPK pathway could upregulate the expression of numerous oncoproteins including chemokines, vascular endothelial growth factor A (VEGFA), matrix metalloproteinases (MMPs), prohibitin, vimentin, MET, NF-κB, HIF1α, EG-VEGF, transforming growth factor-β (TGFβ), and thrombospondin 1 (TSP1) [30]. Previous studies have demonstrated that increased activation of MAPK pathway, which is related with mutations of BRAF gene, is generally found in thyroid cancers [31, 32]. Nucera et al reported that 29-83% of thyroid cancer was accompanied by mutations of the BRAF gene [33]. Xing et al [34] reviewed 29 studies that investigated BRAF^V600E^ variation in thyroid tumor and found that 44% (810/1,856) of PTC and 24% (23/94) of ATC involved BRAF^V600E^ variation, while no BRAF^V600E^ variation was found in the 165 follicular thyroid carcinomas (FTCs), 65 MTCs, and 542 benign thyroid tumors. These findings have suggested that BRAF^V600E^ variation is closely associated with the development of PTC [34]. The findings that BRAF^V600E^ variation is associated with pathological features including bigger PTC diameter, multifocality, extrathyroidal invasion, and lymph node metastasis [35, 36] have suggested BRAF mutation is closely associated with poor prognosis of PTC [37-39]. Vemurafenib, a selective BRAF inhibitor, could effectively be used in treating PTC with BRAF^V600E^ mutation, suggesting that reduction in the activity of MAPK pathway by inhibiting BRAF is effective for treating thyroid cancer [40]. In another study, AZD6244, an MAPK pathway inhibitor, was administered to PTC cell line TPC1, and the proliferation rate of the cells decreased by 70%; treating TPC1 bearing nude mice with AZD6244 also increased the median time to progression to 32 days (the median time to progression was 10 days for the mice in the control group) [41]. In a multicenter clinical study, AZD6244 was used to treat 39 patients with ^131^I refractory PTC (IRPTC). After an evaluation based on the data of 32 patients, one partial response (3%), 21 stable disease (54%), and 11 progressive disease (28%) were found. Disease stability maintenance occurred at 16 weeks in 49% and 24 weeks in 36% of the patients, and the median progression-free survival (PFS) was 32 weeks. The median PFS of the patients with BRAF^V600E^ variation (12/26, 46%) was 33 weeks, which was longer than that in the ones with wide type BRAF (11 weeks); however, the difference was not statistically significant (HR = 0.6, P = 0.3) [42]. MAPK pathway could also be regulated by upstream factors including receptor tyrosine kinases (RTKs), RAS oncogene, and RAF protein-serine/threonine kinases. Tyrosine kinase inhibitors (TKIs) including sorafenib [43-49], pazopanib [50, 51], axitinib [52], sunitinib [53-57], and motesanib [58, 59] could not only inhibit the MAPK pathway, but also inhibit several targets. TKIs have been considered as promising drugs for refractory thyroid cancers. Several ongoing phases I to III clinical trials with these inhibitors have already provided encouraging results. In November 2013, the United States Foods and Drugs Authority (FDA) approved the use of sorafenib for treating radioactive iodine-refractory differentiated thyroid cancer, which made sorafenib the third drug approved for targeted therapy of thyroid cancer (following vandetanib and cabozantinib) (Table 1 [40, 42-93]).

**Table 1:** Drugs Targeting the Signaling Pathways in Thyroid Cancer Studied in Current Clinical Trials

Drug	Targets	Cancer type	Phase of clinical trials	FDA approved	References
Vemurafenib	BRAF	DTC	Phase I-II	-	[40]
AZD 6244 (ARRY-142886, selumetinib)	MEK1/2	DTC	Phase I-II	-	[42, 43, 65, 66]
Sorafeib (BAY43-9006)	VEGFR1-3, PDGFRβ, KIT, RET, BRAF, CRAF, FLT3	DTC, MTC, ATC	Phase I-III	DTC	[43-49, 60, 67]
Pazopanib	VEGFR1-3, PDGFR, KIT	DTC, ATC	Phase I-II	-	[50, 51, 68, 69]
Axitinib (AG-013736)	VEGFR1-3, PDGFRβ, KIT	DTC, MTC, ATC	Phase I-II	-	[52, 70, 71]
Sunitinib (Su11248)	VEGFR1-3, PDGFR, KIT, RET, FLT3, CSF-1R	DTC, MTC	Phase I-II	-	[53-57]
Motesanib (AMG-706)	VEGFR1-3, PDGFR, KIT, RET	DTC, MTC	Phase I-II	-	[58, 59, 72]
Temsirolimus	PI3K/Akt/mTOR	MTC	Phase I	-	[61]
Everolimus (RAD001)	PI3K/Akt/mTOR	PTC, MTC	Phase I-II	-	[62, 63, 73]
Bortezomib	Proteasome	?	Phase I-II	-	[64, 74, 75]
Gefitinib (ZD1839)	EGFR	DTC, MTC, ATC	Phase I-II	-	[76, 77]
Vandetanib (ZD6474)	VEGFR2-3, RET, EGFR	DTC, MTC	Phase I-III	MTC	[78-80]
Cabozantinib (XL-184)	VEGFR2, RET, MET, KIT	DTC, MTC	Phase I-III	MTC	[81-83]
Cediranib	VEGFR	DTC	Phase I	-	[84]
Trametinib (GSK1120212)	MEK1/2	DTC	Phase I	-	[85]
Dabrafenib (GSK2118436)	BRAF	PTC	Phase I-II	-	[86, 87]
Lenvatinib (E7080)	VEGFR1-3, PDGFR, KIT, FGFR	DTC, MTC	Phase I-II	-	[88]
Imatinib (ST1571)	PDGFRα, PDGFRβ, KIT, RET	MTC, ATC	Phase I-II	-	[89, 90]
Combretestatin	Angiogenesis	MTC, ATC	Phase I	-	[91]
PLX-4032 (RG7204)	BRAF	DTC	Phase I	-	[92]
Fosbretabulin	VE-cadherin	ATC	Phase I-II	-	[93]

DTC: differentiated thyroid carcinoma; PTC: papillary thyroid cancer; MTC: medullary thyroid carcinoma; ATC: anapestic thyroid carcinoma; BRAF: B sereine/threonin kinase; VEGFR: vascular endothelial growth factor receptor; PDGFR: platelet-derived growth factor receptor; KIT: stem cell factor receptor; RET: rearranged during transfection; MEK1/2: mitogen-activated protein kinase1/2; FLT3: fms-like tyrosine kinase 3; CSF-1R: colony stimulating factor-1 receptor; EGFR: epidermal growth factor receptor; VE-cadherin: vascular endothelial cadherin; -: not FDA approved for thyroid cancer.

## PI3K/Akt Signaling Pathway

PI3K is a complex present in cytoplasm, which could catalyze the phosphorylation of D3 position of phosphatidylinositol. Protein kinase B (PKB), a protein encoded by retroviral oncogene υ-Akt, is a downstream target protein of PI3K. Phosphorylated Akt (p-Akt), which is the activated form of Akt, could enter cytoplasm or cellular nuclei to phosphorylate a series of substrates and therefore exert biological functions. PI3K/Akt pathway is a very important intracellular signaling pathway for mammals, which is closely associated with cell proliferation, transformation, metabolism, motility, and development and progression of tumors [94]. Activated PI3K/Akt pathway could also activate other pathways including Wnt-β-catenin, HIF1α, FOXO3, and NF-κB pathways [95-97]. PI3K-activated Akt could in turn phosphorylate a series of downstream target proteins including Bad, caspase 9, forkhead, Par-4, p2l, and mammalian target of rapamycin (mTOR) to activate or inhibit their functions, which finally promote cell survival. Therefore, Akt has been regarded as an antiapoptotic regulator [98]. mTOR is also a member of PI3K protein kinase family and an important downstream molecular of PI3K pathway. PI3K/Akt/mTOR pathway could induce the development of tumor via various pathways; for instance, activation of mTOR could inhibit autophagy as a critical regulator in the initial period of autophagy. Activated Akt could inhibit the activation of caspase 3 and caspase 9 by phosphorylate Ser 196 and thus inhibit apoptosis; PI3K/Akt pathway could inactivate Bax by phosphorylate Ser 184 to inhibit apoptosis, and PI3K/Akt could induce phosphorylation of FOXO-1, which could enter cellular nuclei and expression to regulate cell cycle [99-102]. PI3K/Akt pathway could also promote tumor metastasis by promoting cell motility and angiogenesis; for instance, activated Akt could increase the activity of NF-κB and thus increase the motility of tumor cells. Activation of p70^s6k^, a protein downstream of mTOR, could increase cell motility. PI3K/Akt pathway could upregulate the expression of MMP-2 mRNA and protein, which could degrade extracellular matrix and promote tumor metastasis and invasion. PI3K/Akt could upregulate the expression of HIF1α and induce the transcription of VEGF gene to increase the expression of VEGF, which in turn induce angiogenesis and increase blood supply of tumors [103, 104]. RAS mutation is the second most gene mutation in thyroid cancer, which mainly activates PI3K/Akt pathway [105, 106]. Studies have reported that about 93% of FTCs and 96% of ATCs have mutations of the genes that encode proteins involved in PI3K/Akt pathway. These mutations include mutations of RTK, RAS, and phosphatase and tensin homolog deleted on chromosome 10 (PTEN) encoding genes, extra copies of phosphoinositide-3 kinase catalytic α (PIK3C A), phosphoinositide-3 kinase catalytic β (PIK3C B), 3-phosphoinositide-dependent kinase-1 (PDK1) encoding genes, and rearrangement of peroxisome proliferators-activated receptor γ/paired box gene 8 (PPARγ/Paχ8) encoding gene [107]. Xing et al suggested that activation of MAPK pathway could increase the development of PTC, which could further progress to ATC after dedifferentiation induced by activated PI3K/Akt pathway; in addition, PI3K/Akt pathway activation could induce the development of FTC, which could also progress to ATC after the induction caused by further activated PI3K/Akt pathway and/or MAPK pathway [108]. Administration of rapamycin (an mTOR inhibitor) to PTC1 could reduce the proliferation rate of the cells by 80%; animal study also demonstrated that rapamycin could increase the median time to progression to 23 days (the median time to progression was 10 days for control group) in TPC1 bearing nude mice [41]. Similarly, GDC-0941, an inhibitor of PI3K/Akt pathway could inhibit the compensatory survival response of thyroid cancer cells by arresting the cells at G1 phase and finally increase cell apoptosis [109]. Except for PI3K pathway, GDC-0941 could also inhibit HIF-1α pathway to reduce the metastasis of thyroid cancer [110]. Currently, many drugs including temsirolimus, everolimus, rapamycin, INK-128, OSI027, AZD8055, GSK690693, MK-2206, CAL-101, BYL719, PX866, GDC0941, BKM120, and ZSTK474 that target PI3K/Akt have been investigated in phases I to III clinical trials [111], among which, temsirolimus and everolimus have been found with remarkable efficacies in treating thyroid cancers [61-63].

## NF-κB Signaling Pathway

NF-κB is a family of proteins that are homo- or heterodimers of Rel family members. Five NF-κB/Rel family members including Rel (cRel), p65 (RelA, NF-κB3), RelB, p50 (NF-κB1), and p52 (NF-κB2) have been identified in mammalian cells. IκB, another family of protein consisting of IKKα, IKKβ, IKKγ, IKKδ, IKKε, and BCL-3 that could inhibit the activity of NF-κB, has also been identified in cytoplasm. IκB mainly combines with NF-κB/Rel proteins in cytoplasm to keep them sequestered. Extracellular signals could activate IκB kinase (IKK) to phosphorylate IκB and degrade IκBα; the released p50/p65 dimer then enters the cell nuclei to combine with special sequence of κB and in turn induce the transcription of target genes [112, 113]. The association between NF-κB and development of tumor mainly relies on the fact that NF-κB could inhibit cell apoptosis. For instance, NF-κB could influence cell apoptosis by regulating cytokines including TNF-α, IL-1β, IL-6, and IL-8; it could also inhibit apoptosis by inducing or upregulating the expression of antiapoptotic gene such as bcl-2 or by inducing the expression of TNF-α receptor family (TRAF1 and TRAF2), cellular inhibitor of apoptosis proteins (c-IAP1 and c-IAP2), and zinc finger protein A20 [114, 115]. Previous studies have demonstrated that NF-κB pathway is activated in several malignancies including leukemia, breast cancer, pancreas cancer, and thyroid cancer [116-120]. Specifically for thyroid cancer, NF-κB activation was found in PTCs, FTCs, and ATCs, and researchers suggested that activation of NF-κB could promote dedifferentiation of PTCs and FTCs and thus play important roles in each stage of thyroid cancer [119-122]. Activation of NF-κB could upregulate the expression of COX-2, IL-8, and GST-π in FTCs [120]. BRAF^V600E^ could increase the carcinogenicity of thyroid cancer cells through NF-κB mediated c-IAP1, c-IAP2, and XIAP, thus increasing the invasiveness of thyroid cancer cells [123, 124]. Yamashita et al [125] suggested that mutations of BRAF and RAS genes and rearrangement of RET/PET gene could activate MAPK pathway, which in turn activate NF-κB pathway and increase the progression and invasiveness of PTCs. NF-κB could regulate the expression of MMPs, urokinase-type plasminogen activator (uPA), and IL-8 to allow cancer cells obtain invasiveness, which could then infiltrate surrounding tissues and metastasize to distant organs. Specifically, MMPs could dissolve and damage extracellular matrix and thus increase the infiltration and metastasis of cancer cells. Komorowski et al demonstrated that level of MMP-2 was significantly increased in PTC, and levels of MMP-3 and MMP-9 were significantly increased in MTC, suggesting that NF-κB activation could increase the invasiveness and metastasis of thyroid cancer cells [126]. Inhibition of NF-κB activation could affect the growth, apoptosis, and invasion of thyroid cancer cells [127]. In addition, activation of NF-κB could be inhibited when the activities of IKKs and proteasome are inhibited, or p50/p65 dimers are prevented from entering cell nuclei or combining with target DNA [112, 113]. Small-molecule triptolide, an NF-κB inhibitor, was used to treat two human ATC cell lines, namely TA-K cells and 8505C cells. The expression of cyclinD1, VEGF, and uPA was effectively inhibited, which in turn effectively reduced the angiogenesis and invasion of ATC [122]. Bortezomib, an inhibitor of proteasome, could increase the expression of p21(CIP1/WAF1) and thus inhibit cell growth, increase cell apoptosis, and arrest cells at G2-M phase in two ATC cell lines, namely C643 and SW1736 cells [128]. Currently, bortezomib has been approved by FDA to treat myeloma; however, more clinical studies are needed to evaluate the efficacy and safety of treating thyroid cancer with bortezomib [64].

## TSH Receptor (TSHR) Signaling Pathway

TSHR is a guanine nucleotide-binding G protein-coupled receptor, which could combine with TSH and thus stimulate the growth of thyrocytes directly or indirectly by stimulating growth factors including autocrine growth factors and VEGF; in addition, sodium iodide symporter (NIS) promoter and upstream enhancers could also be activated and thus upregulate the expression of NIS, which could in turn increase the transport of iodide to cellular membrane [129]. Two intracellular signaling pathways, namely Gsα-mediated adenylyl cyclase-cyclic AMP (cAMP) signaling pathway and Gq- or G11-mediatedphospholipase C β-inositol 1,4,5-trisphosphate-intracellular Ca^2+^ signaling pathway, could be activated after the combination of TSHR with TSH [7]. Each signaling molecule in these pathways could interact with the signaling molecules of other pathways including Wnt, PI3K, and MAPK pathways to form a network [130]. Liu et al investigated 34 paraffin-embedding specimens of classical PTCs and 39 PTCs with tall-cell features, and they found that the positive expression rate of TSHR was significantly lower in PTCs than in the normal thyroid tissues adjacent to the cancers (χ^2^ = 15.70, P < 0.05) and lower in PTCs with tall-cell features than in classical PTCs (χ^2^ = 4.24, P < 0.05) [131]. Other studies also demonstrated that mRNA levels of NIS and TSHR were significantly lower in thyroid cancer tissues than in benign thyroid nodules or normal thyroid tissues [132]. TSHR expression could be very low in DTC and even undetectable in poorly differentiated carcinoma of the thyroid [133, 134]. During treatment or natural course, TSHR expression and iodide uptake could decrease in about 2-5% of DTCs, degenerative changes of the morphology and functions of thyroid cancer cells could also occur, and the cells could then lose their differentiated features and become insensitive to radioiodine therapy and TSH suppressive therapy [5]. Xing et al [135] suggested that decrease or lack of TSHR expression in human thyroid cancer tissues or thyroid cancer cell lines could be caused by the methylation of the gene promoter. DNA methylation is an epigenetic change which mainly occurs at the cytosine at or near the promoter of genes, which could regulate the transcription of these genes and cause gene silence, and it could thus decrease or totally inhibit the expression of corresponding protein [136]. Till date, over 25 mutations have been identified on TSHR gene [137, 138]. Clinical and *in vitro* studies have demonstrated that retinoic acid could induce redifferentiation of thyroid cancer cells, increase the expression of NIS and uptake of radioactive iodine, and therefore increase the efficacy of radioiodine therapy [139, 140]. Histone deacetylase inhibitor could increase the expression of NIS gene through posttranscriptional modification. Triehostatin A (TSA), a histone deacetylase inhibitor, could significantly increase the expression of NIS, induce the redifferentiation, and restore ^131^I uptake in three thyroid cancer cell lines (TPC-1, XTC-1, and FTC-133) [141]. Single or combined use of RDEA119 (a MAPK pathway inhibitor) and perifosine (a PI3K/Akt pathway inhibitor) could increase the expression of iodide-handling genes in 11 thyroid cancer cell lines. Additional use of SAHA (a histone deacetylase inhibitor) could increase the expression of NIS, TPO, and TSHR genes by about 2,500, 1,000, and 600 folds, respectively. ^125^I uptake was increased by three, five, and four folds for K1, C643, and KAT18 cells, respectively [142]. Sunitinib could also increase the expression of NIS gene by inhibiting MEK/ERK and SAPK/JNK pathways in PTCs [143]. These findings demonstrated that TSHR could collaborate with other pathways to form a network, and inhibition of TSHR pathway could increase the activity of other pathways, which could increase the growth of cancer cells, decrease the expression of NIS, and thus reduce iodide uptake of cancer cells. Therefore, targeting TSHR is an optimal method to induce the redifferentiation of poorly differentiated carcinoma of the thyroid and increase the iodide uptake of iodine-refractory thyroid carcinomas by increasing the NIS expression [144].

## Wnt-β-Catenin Signaling Pathway

Wnt pathway is named after Wnt protein, a protein that initiates this pathway. It is associated with cell development and differentiation. Wnt pathway includes canonical Wnt pathway (Wnt-β-catenin pathway) and non-canonical pathway (Wnt-Ca^2+^ or Wnt-NK pathway) [145]. When activated, Wnt could combine with its receptor Fzd to activate Dsh protein, which is located inside the cells. Phosphorylated Dsh protein could then transduce the signals into the cells to inhibit the activity of the complex made up of APC, GSK-3β, Axin, and β-catenin, which could cause the accumulation of β-catenin in the cells. β-catenin could enter the cell nuclei and combine with transcription factors of Tcf/LEF family to form a complex, which could activate the transcription of downstream target genes including c-myc and cyclin D1 to increase the differentiation and proliferation of tumor cells [146]. Kurihara et al investigated the associations between Wnt-β-catenin pathway and ATC, and they found that β-catenin expression could be detected in 40.9% of the cell nuclei and 63.6% of the cytoplasm for the 22 patients with ATC; mutation rate of β-catenin, APC, and Axin1 genes were 4.5%, 9.0%, and 81.8%, respectively. The expressions of cyclin D1 and c-myc, two targets of Wnt-β-catenin pathway, were as high as 27.3% and 59.1%, respectively, suggesting that changes of Wnt-β-catenin pathway could be associated with the development of ATC [147]. Wnt-β-catenin could also regulate the expression of cyclin D1, which is associated with lymph node metastases in PTCs [148]. Interactions between β-catenin and E-cadherin could affect cell adhesion. Studies showed that E-cadherin could combine with cytoplasmic β-catenin to form E-cadherin/β-catenin/α-catenin complex, which in turn combines to cortical actin cytoskeleton to maintain cell stability and polarity of during adhesion that is essential for the integrity and function of epithelial tissues. Downregulation or absence of cell adhesion molecules including E-cadherin could induce the changes from keratin-dominant cytoskeleton to vimentin-dominant cytoskeleton, which could change the cell morphology, increase the motility of the cells, and thus increase the risk of tumor metastasis. Overexpression of E-cadherin could block the transcription ability of β-catenin, effectively shut-down the expression of target genes, and thus inhibit the proliferation and migration of the cells [149]. Dickkopf-1, a Wnt-β-catenin pathway inhibitor, was used to treat human PTC cell lines (including SNU-790, B-CPAP, and BHP10-3). Dickkopf-1 could effectively inhibit the survival and migration of PTC cells by regulating Wnt-β-catenin pathway and E-cadherin expression [150]. Antisense oligonucleotides, RNA interference, and neutralizing antibodies could be used for the targeted inhibition of Wnt-β-catenin pathway [151]. Rao et al treated ATC cell lines with imatinib mesylate, a specific tyrosine kinase inhibitor, and they found that imatinib mesylate could reduce the expression of β-catenin, stabilize the β-catenin/E-cadherin complex, and decrease the invasiveness of thyroid cancer; therefore, imatinib mesylate could be used as a drug for targeted therapy for some patients with ATC [152].

## Notch Signaling Pathway

Notch pathway is composed of receptor, ligand, and intracellular effector CSL (CBF1/RBP-Jκ, Su(H), Lag1). Combination of Notch ligand and receptor could trigger two proteolyses. As a result of proteolysis, the Notch receptor then enters the nuclei and combines with downstream CSL to form a transcriptional activation complex and initiate transcription of target genes. Notch pathway could not only inhibit the differentiation of T cells and granulocyte, neurogenesis, and muscle formation, but also directly induce the ordered cell differentiation [153, 154]. Dysfunction of Notch pathway is associated with several biological processes including cellular function, microenvironment, cell proliferation, apoptosis, adhesion, epithelial-mesenchymal transition, and angiogenesis that are related to development of numerous tumors. Except for directly inducing the development of tumor, Notch pathway could also interact with many other pathways and thus indirectly induce the development of tumors [155]. Cancer treatments that target Notch pathway include γ-secretase inhibitors (GSIs), antibodies to Notch receptors, and Notch-1 siRNA. Previous clinical trials have demonstrated that monoclonal antibodies have shown efficacy against metastatic thyroid cancer, non-small cell lung cancer, sarcoma, colorectal cancer, melanoma, and ovarian cancer with GSIs [156]. Notch signal could be expressed in normal thyroid tissues and adenomas but not in ATCs. Overexpression of Notch signal could induce redifferentiation of thyroid cancer cells, which could reduce the growth of the cancer cells and directly affect NIS promoter to increase the expression NIS. This suggests that Notch pathway plays a role in the development, progression, and differentiation of thyroid cancer [157]. Activation of Notch pathway could effectively inhibit the cell growth of ATC *in vivo* and *in vitro*, and it inhibited the growth of MTC *in vivo* [158, 159]. Valproic acid was used to activate Notch 1 signal to induce apoptosis and inhibit cell growth in MTC cells, which provided experimental evidence for using such drugs in treating advanced MTCs [160]. Patel et al treated ATC cell line HTh7 with hesperetin, a Notch pathway activator, and they found that hesperetin could induce the expression of TTF1, TTF2, paired box gene 8, TSHR, and NIS; it thus improved the redifferentiation of ATC cells and inhibited ATC cell proliferation by inducing apoptosis [161]. In summary, Notch signaling pathway is closely associated with the development of thyroid cancer, and it could be an important target for treating thyroid cancer.

## Other Signaling Pathways

Some other signaling pathways are also associated with the development and progression of thyroid cancer. For instance, RASSF1-MST1-FOXO3 pathway is associated with BRAF^V600E^ gene mutation [162], C-met pathway is associated with the growth, invasion, and lymph node metastasis of thyroid cancer [163, 164], and sonic hedgehog pathway is ubiquitously expressed in thyroid cancers and is associated with the development, staging, and lymph node metastasis of thyroid cancer [165].

In summary, each of the signaling pathways could exert its function singly or through network with other pathways. These pathways could cooperate, promote, antagonize, or interact with each other to form a complex network for the regulation. Dysfunction of this network could increase the development, progression, invasion, and metastasis of thyroid cancer. Signaling pathway-related targeted therapy could be the fourth treatment method following surgical removal, radioiodine therapy, and endocrine suppressive therapy. In addition, it could induce the redifferentiation of residual thyroid cancer to achieve “benign” change, and performing immune induction to improve the ability against cancer is also very important in treating thyroid cancer.
